# STRONGHOLD first-year results of biomechanically calculated abdominal wall repair: a propensity score matching

**DOI:** 10.1007/s10029-023-02897-7

**Published:** 2023-10-10

**Authors:** C. Lesch, R. Nessel, D. Adolf, M. Hukauf, F. Köckerling, F. Kallinowski, For the STRONGHOLD/Herniamed-Collaborators GROUP, A. Willms, R. Schwab, K. Zarras

**Affiliations:** 1 General, Visceral and Transplantation Surgery, University Hospital Heidelberg, Im Neuenheimer Feld 410, 69120 Heidelberg, Germany; 2 General, Visceral and Pediatric Surgery, Klinikum Am Gesundbrunnen, Am Gesundbrunnen 20‑26, 74078 Heilbronn, Germany; 3 StatConsult, Am Fuchsberg 11, 39112 Magdeburg, Germany; 4 Vivantes Humboldt Hospital Berlin, Center for Hernia Surgery, Academic Teaching Hospital of Charité University Medicine, Am Nordgraben 2, 13509 Berlin, Germany; 5 General and Visceral Surgery, Bundeswehrkrankenhaus Hamburg, Lesserstrasse 180, 22049 Hamburg, Germany; 6 General, Visceral and Thorax Surgery, BundeswehrZentralkrankenhaus Koblenz, Rübenacher Strasse 170, 56072 Koblenz, Germany; 7 Visceral, Minimal Invasive and Oncological Surgery, Marien Hospital Düsseldorf, Schloßstraße 85, 40477 Düsseldorf, Germany

**Keywords:** Incisional hernia, Biomechanically calculated incisional hernia repair, Abdominal wall reconstruction, Propensity score matching for incisional hernia repair, STRONGHOLD, Herniamed

## Abstract

**Purpose:**

Every year around 70,000 people in Germany suffer from an abdominal incisional hernia that requires surgical treatment. Five years after reconstruction about 25% reoccur. Incisional hernias are usually closed with mesh using various reconstruction techniques, summarized here as standard reconstruction (SR). To improve hernia repair, we established a concept for biomechanically calculated reconstructions (BCR). In the BCR, two formulas enable customized patient care through standardized biomechanical measures. This study aims to compare the clinical outcomes of SR and BCR of incisional hernias after 1 year of follow-up based on the Herniamed registry.

**Methods:**

SR includes open retromuscular mesh augmented incisional hernia repair according to clinical guidelines. BCR determines the required strength (**C**ritical **R**esistance to **I**mpacts related to **P**ressure = CRIP) preoperatively depending on the hernia size. It supports the surgeon in reliably determining the **G**ained **R**esistance, based on the mesh-defect-area-ratio, further mesh and suture factors, and the tissue stability. To compare SR and BCR repair outcomes in incisional hernias at 1 year, propensity score matching was performed on 15 variables. Included were 301 patients with BCR surgery and 23,220 with standard repair.

**Results:**

BCR surgeries show a significant reduction in recurrences (1.7% vs. 5.2%, *p* = 0.0041), pain requiring treatment (4.1% vs. 12.0%, *p* = 0.001), and pain at rest (6.9% vs. 12.7%, *p* = 0.033) when comparing matched pairs. Complication rates, complication-related reoperations, and stress-related pain showed no systematic difference.

**Conclusion:**

Biomechanically calculated repairs improve patient care. BCR shows a significant reduction in recurrence rates, pain at rest, and pain requiring treatment at 1-year follow-up compared to SR.

## Introduction

Incisional ventral hernias occur frequently after abdominal surgeries. They can also occur in physically active people or after pregnancy. Hernia patients can experience pain and loss of their physical strength. This leads to unemployment, social withdrawal, depression, and great medical needs [3, 4]. In the US, more than $7 billion US is spent each year on incisional hernia repair, sick leave, and early retirement [5]. In Germany, the cost amount is at least € 1.8 billion. In the United States, more than 100,000 patients require hernia surgery each year [5]. However, the recurrence rate after surgery remains high [6]. The care and clinical practice of hernia surgery varies widely with different techniques and guidelines [7, 8]. A tailored approach to hernia surgery is recommended by hernia societies [7]. However, even when tailored by experts, individualized repair often fails [9].

In 2013, repetitive pressure impacts were recognized as a destructive force leading to incisional hernia formation [10, 11]. The durability of reconstructions can be determined by subjecting reconstructed tissues to cyclic pressure impacts on a test bench. Each material used, e.g., mesh and fixation, can be assigned a specific holding force [1, 12]. It has been found that considering the mesh–defect area ratio (MDAR) allows for a more reliable reconstruction than considering the mesh overlap alone [13, 14]. This provides the basis for the development of a biomechanically driven repair concept, the **G**ained/**C**ritical **R**esistance to **I**mpacts related to **P**ressure (GRIP/CRIP) concept. It includes two mathematical formulas to allow for individual hernia reconstruction. The **CRIP** formula determines the required durability of a reconstruction based on the defect size and tissue characteristics. The formula results in a numerical value that must be reached by an individual reconstruction to be durable. Larger hernias, extensible tissue, and repeated repairs require a stronger reconstruction, i.e., higher CRIP values. The **GRIP** formula advises the surgeon whether the selected reconstruction will achieve a sufficient stability for the individual patient. It allows the surgeon to fill in the selected reconstruction technique and materials and assigns them numerical factors. Then, the algorithm determines whether the stability achieved by the repair (GRIP) reaches the necessary stability (CRIP). Once the GRIP exceeded the CRIP value, the tested reconstructions withstood hundreds of impulse loads [1]. The GRIP/CRIP concept satisfies the need for personalized hernia repair. It enables biomechanically calculated repair (BCR) by determining the minimum force required for abdominal wall repair (CRIP) in each patient and assists the surgeon in achieving reliable and durable abdominal wall reconstruction (GRIP) [1]. Tissue distension of each patient’s abdominal wall was determined by clinical assessment of stability. Unstable abdominal walls were analyzed with CT scans at rest and during a Valsalva maneuver, allowing for individualization of complex hernia repair with excellent results at 1 year (Kallinowski, 2021a #77).

The STRONGHOLD registry was established as a subgroup of the Herniamed registry to evaluate the outcome of biomechanically calculated reconstruction (BCR) of the abdominal wall. STRONGHOLD consists of nine German hospitals where hernia care is performed according to the GRIP/CRIP concept [15]. This manuscript represents the first clinical application of the GRIP/CRIP concept by a larger group of surgeons.

After 1 year of follow-up, we compared the outcome of the BCR outcome with standard repair (SR) within the Herniamed registry. The comparison included intraoperative, postoperative, and overall complications, complication-related reoperations, and pain and recurrence rates at 1 year. We used propensity score matching. This statistical method forms control groups for a sample from a larger heterogeneous control population. It allows control subjects with similar characteristics to the study population to be found for comparison on an outcome variable.

## Methods

### Herniamed and STRONGHOLD

Herniamed is a German internet-based registry for inpatient and outpatient hernia surgery. It aims to monitor and evaluate important outcome data and improve the quality of patient care for all types of hernia surgeries. All interested hospitals and surgeons can easily enter data on all their hernia surgeries according to a scientifically validated standard procedure with the patient’s consent [16]. STRONGHOLD follows the same principles as a subset of Herniamed. It collects additional data for the biomechanically calculated reconstruction.

### Standard repair

Standard repair includes open retromuscular mesh placement performed, which is without consideration of the BCR concept. SR monitors 30 patient- and hernia-related factors, such as risk factors, previous surgeries, and hernia size. The general recommendations (e.g., from the European Hernia Society [7]) for hernia repair are well known and largely reflected in the surgical expertise available in certified hernia centers.

### Biomechanically calculated repair (BCR) in theory and practice

The aim of the BCR is to balance the forces acting on the abdominal wall and the retaining forces of the reconstructed tissue. The abdominal cavity is supported by several layers of connective and muscular tissue. The abdominal wall must be able to withstand the weight of the internal organs and additional physiological loads. The total intra-abdominal pressure can be understood as a hydraulic system influenced by gravity, compression, and shear deformation [17]. Coughing, jumping, or weight lifting increases the intra-abdominal pressure [18, 19]. The abdominal wall needs to be sufficiently stable to withstand these forces. During the initial period after a repair, a reconstruction adapts to the mechanical influences with an elastic–plastic deformation. This process determines the long-term durability of the reconstruction. Repeated microplastic deformations can add up to a large plastic deformation. Recurrence begins with each microplastic deformation but only becomes apparent over time [20–22]. In contrast, an elastic deformation pattern allows healing [23].

All surgeons contributing to Herniamed are interested, well-educated surgeons. Compared to surgeons contributing only to Herniamed, BCR surgeons document seven additional items to the thirty factors considered by the SR: the MDAR, the shape of the mesh, the minimal overlap, the kind of fixation elements, the amount of fixation elements, the suture closure of the defect, and the peritoneal closure. At Heidelberg University Hospital, an assessment of tissue elasticity is added as a sixth factor (as explained below). An algorithm calculates CRIP and GRIP values for all hernia repairs from the STRONGHOLD data, as explained below.

The calculations leading to the BCR are based on more than 240 preclinical studies performed on porcine and bovine tissues. The concept was developed in more than 2000 experiments over a period of 10 years. For this purpose, bovine and porcine tissues were systematically reconstructed with different meshes and sutures using different techniques [1, 24]. Afterwards, the reconstructed tissues were then tested on a test bench, which loaded the tissues with 425 repetitive pressure impacts of approximately 210 mmHg [25]. From these preclinical results, factors were calculated to estimate the durability of each reconstruction. The results were then gradually incorporated into clinical practice.

**Fig. 1:**
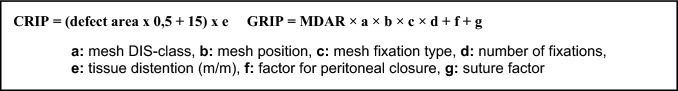
CRIP formula for calculating the necessary stability**. **GRIP formula with factors for calculating the achieved stability [1, 2]. MDAR = mesh–defect area ratio. The letters indicate the coefficient for the material and/or technique used

The BCR formula is based on the ratio of the defect size to the size of the implanted mesh (MDAR) [13]. The MDAR formula has been extended to include several biomechanical factors, which influence the durability of an abdominal wall repair. This extension represents the GRIP/CRIP concept. The Critical Resistance to Impacts related to Pressure (CRIP) provides a factor for the strength required to achieve a durable repair. It is a function of hernia size and patient tissue characteristics (e). The acronym GRIP describes the gained resistance of the repair to pressure effects. The GRIP formula includes several biomechanical factors that influence the durability of a customized mesh repair [1, 2]. It is based on the MDAR and considers the hernia area, various mesh (a–d), and suture factors (f, g). The separate factors are broken down in Fig. 1 and Table 1 and explained below.

**Table 1:** Overview of the GRIP factors in detail with their numerical value known to date

Factor	Meaning			Number value
MDAR	Mesh-area ÷ defect area			Varying
a	Mesh DIS-class	**A:** DynaMesh^®^, Progrip^®^		1
		**B:**	Ultrapro^®^ Advance	0.5
			Optilene^®^	0.4
		**C:**	Ultrapro^®^	0.25
			Adhesix^®^	0.1
b	Mesh position	Sublay		1
		Onlay		0.5
		IPUM/Underlay		0.9
c	Mesh fixation typed	Sutures, Protack^®^, Securestrap^®^		0.5 per fixation
		Absorbatack^®^, Glubran^®^		0.33 per fixation
		Fibrin glue		0.15 per fixation
d	Number of mesh fixations			n
e	Tissue distention (m/m)			1 – **∞**
f	Peritoneal closure			4
g	Suture factor			0.5 per suture for correct horizontal + 0.5 for correct vertical placement = 1 per precise suture

Preoperatively, the surgeon calculates the CRIP according to the patient’s individual defect size and tissue quality. The area of the hernia orifice is assessed clinically or measured on a CT scan.

After assessing the CRIP, the surgeon selects an appropriate customized reconstruction based on the GRIP formula. The calculations are performed by an algorithm based on the data provided by the surgeon in the STRONGHOLD registry. For better understanding, the algorithm is explained here.

First, the mesh area is divided by the defect area, giving the MDAR. The mesh reconstruction is then determined using four parameters (a–d). Factor a describes the adhesiveness of a particular mesh type to the tissue, which was assessed by evaluating the mesh displacement during repetitive loading. Different meshes have different adhesion properties to the surrounding tissue. This is defined by the DIS-class (a). Highly gripping meshes are classified as DIS-class A meshes, and less gripping meshes are classified as DIS-class B or C [25]. For factor b, three mesh positions in the porcine abdominal wall were investigated for the highest reconstruction stability. Sublay mesh placement provides a higher stability than onlay or underlay placement (Table 1) [10, 26]. Factors c and d were obtained by evaluating various mesh fixation techniques and devices. Different types of mesh fixation provide different ranges of stability, adding 0.15–0.5 to the GRIP with each fixation point (d). Factor e was only analyzed in detail in Heidelberg using a CT scan. The other Stronghold clinics do not perform imaging in every suspected patient.

If there is clinical suspicion of a highly distensible abdominal wall (> 20% or > 15 mm ventral shift), we recommend additional imaging to further clarify the instability [15, 27]. A preoperative CT scan of the patient’s abdomen a rest and during Valsalva maneuver can enhance the tailored approach. It provides crucial information about the behavior of the hernia orifice under stress and can identify unstable zones in the abdominal wall, that require additional reinforcement to ensure a stable, biomechanically calculated repair [15, 27]. The patients’ tissue extensibility in % (multiplication factor “e”) represents potential instability. It is determined by dividing the hernia diameter measured on the CT scan during the Valsalva maneuver by the hernia diameter at rest. The scan must be analyzed at least 12 times with three individual assessments to reduce inter- and intraindividual variation to less than 5%. Half of our patients show tissue distensions of more than 20% during a Valsalva maneuver, as it is performed in Heidelberg [27]. In this case, it is advisable to increase the CRIP value by a factor (e) of 1.2. Elastic tissue requires a stronger reconstruction [15, 26].

Factor f was added, since the closure of the peritoneum adds stability by providing an additional layer of support and a larger mesh-tissue interface [12]. Suturing the defect (factor g) adds strength to the reconstruction depending on its precision. Precisely placed sutures in the horizontal and vertical planes can increase the GRIP by a factor of 1 per stitch [24]. Standardized suturing may be included in BCR but is currently not part of the Stronghold registry. Stronghold surgeons are closely following the guidelines, using a running small-stitch-small-bite suture aiming for a suture-to-wound-length-ratio greater than 4:1.

Surgeons performing BCR-based hernia reconstruction were trained in a seminar that included personal coaching and a ten-page introduction to the topic and a four-page application guide. Additional coaching via telephone or Zoom was available to facilitate the use of BCR. All surgeons are free in their choice of technique. It is not required to store all kinds of materials mentioned above. The concept allows every surgeon to choose a combination that suits them, their hospital and the patient. None of the cases included in this study was bridging documented as the chosen closure technique.

### Clinical evaluation: propensity score matching

We conducted a matched-pair analysis to compare the outcome of incisional hernia surgery considering BCR with SR surgery based on prospectively collected data of the Herniamed database. Included were fully documented elective incisional hernia surgeries by open—sublay or component separation using approved meshes with valid GRIP and valid MDAR. The surgeries had to be performed before 01/01/2021 including 1-year follow-up in patients with valid minimum age of 16 years.

After confirmation of the inclusion criteria and univariable explorative statistics of the patient population, a 1:1 pairwise matching was performed.

Thirteen matching variables were defined to form the propensity scores: BMI (kg/m^2^), age (years), mesh size/defect size ratio, ASA-Score (I/II/III–IV), EHS-Classification (medial/lateral/combined), defect size (W1 < 4 cm/W2 > = 4 – 10 cm/W3 > 10 cm), preoperative pain (yes/no/unknown), and presence of risk factors (without immunosuppression) (yes/no), as well as gender (m/f), recurrence (yes/no), surgical procedure (open-sublay/component separation), immunosuppression (yes/no), and fixation (yes/no). The last five variables are fixed matching variables, which means that no deviation between matched patients is allowed in each case.

Risk factors are present when at least one of the following factors is applicable: COPD, diabetes, aortic aneurysm, cortisone, smoking, coagulopathy, antiplatelet drugs during the last 7 days, and coumarin derivatives.

The robust greedy algorithm (with a caliper of 0.2 standard deviations) was used to assign the elements from the BCR population to appropriate cases of the SR population.

After matching, the balance of the matched samples was assessed using standardized differences. A good balance with respect to the included variables is ensured with a standardized difference of less than 10% (< 0.1). The results are illustrated in Tables 4 and 5. The examined outcome parameters were: intraoperative, postoperative, and general complications, consecutive reoperations, as well as pain at rest and at movement, pain requiring treatment, and recurrences after 1-year follow-up.

The exact McNemar test was performed for testing for a systematic deviation between the comparison groups (BCR vs. SR) with respect to an outcome parameter. An odds ratio adjusted for matched samples (95% confidence interval) was additionally provided. All analyses were performed using SAS 9.4 software (SAS Institute Inc., Cary, NC, USA) and are deliberately considered at the full 5% significance level, i.e., no adjustment for multiple testing is applied and any *p* value ≤ 0.05 corresponds to a significant result.

## Results

**Fig. 2:**
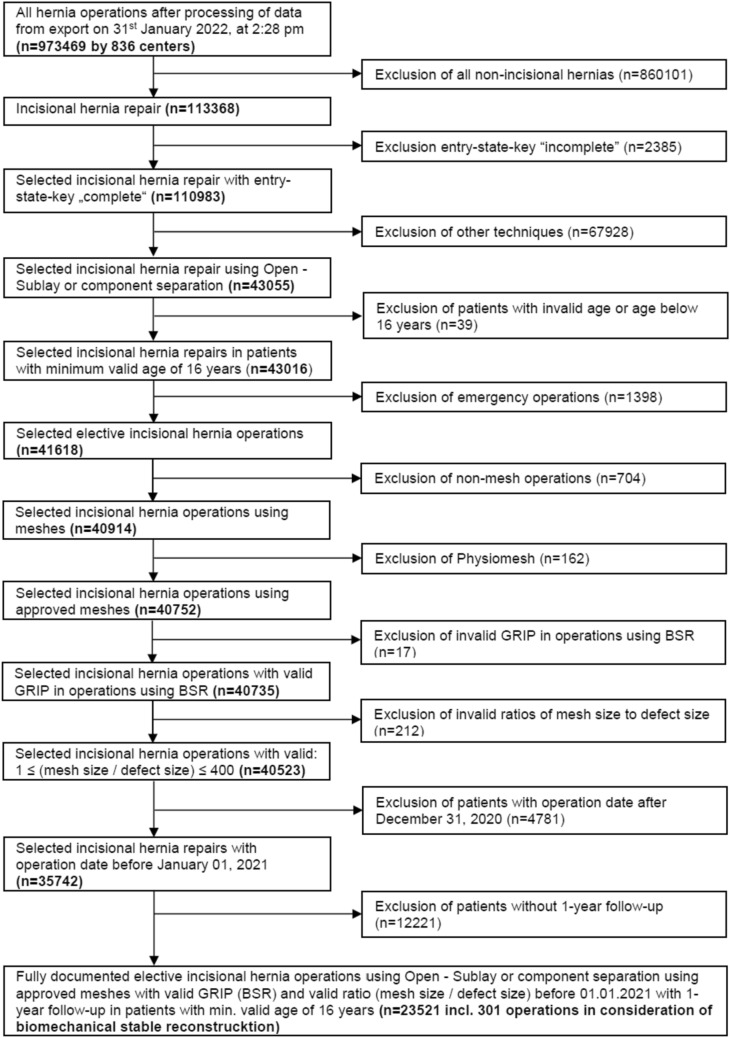
Flowchart of patient inclusion for analysis

The patients were selected as shown in Fig. 2. After patient selection, 301 patients were found that had undergone BCR hernia surgery and 23,220 patients that had a standard repair as shown in Table 2.

**Table 2:** Distribution of frequency of operations with and without BCR consideration

BCR	*N*	%
Yes	301	1.28
No	23,220	98.72
Total	23,521	100.00

The 301 patients underwent BCR hernia surgery in one of the nine hospitals that participate in the STRONGHOLD study. The distribution of BCR surgeries among the STRONGHOLD clinics is shown in Table 3.

**Table 3:** Frequency distribution of hernias operated with consideration of BCR within the centers participating in the STRONGHOLD study

Stronghold-clinic	Procedures	
	*n*	%
1	75	24.9
2	44	14.6
3	40	13.3
4	36	12.0
5	34	11.3
6	34	11.3
7	21	7.0
8	15	5.0
9	2	0.7

To investigate whether systematic differences in outcomes between surgeries with and without consideration of biomechanically calculated reconstruction can be found, matched pairs of patients with and without BCR were formed. Propensity score matching resulted in 291 matched pairs. Accordingly, 96.7% of BCR hernia surgeries could be matched to a similar SR case.

Standardized differences of less than 0.1 are found for all variables in the matched samples. Thus, the matched samples are relatively balanced and can be used for analyses without further adjustment for covariables.

An overview over the patient characteristics with standardized differences before and after matching is provided in Table 4.

**Table 4:** Standardized differences of the stable matching parameters before and after matching

	BCR					
	Yes		No		Standardized differences	
	*n*	%	*n*	%	Matched sample	Original sample
Open—sublay*	196	67.4	196	67.4	0.000	0.752
Component separation*	95	32.6	95	32.6	0.000	0.752
Male*	160	55.0	160	55.0	0.000	0.065
ASA score I	19	6.5	15	5.2	0.059	0.092
ASA score II	153	52.6	167	57.4	0.097	0.113
ASA score III–IV	119	40.9	109	37.5	0.070	0.166
Medial	262	90.0	261	89.7	0.011	0.126
Lateral	76	26.1	74	25.4	0.016	0.173
Combined	47	16.2	44	15.1	0.028	0.347
Defect size W1 (< 4 cm)	62	21.3	68	23.4	0.050	0.081
Defect size W2 (> = 4–10 cm)	134	46.0	123	42.3	0.076	0.190
Defect size W3 (> = 10 cm)	95	32.6	100	34.4	0.036	0.286
Fixation*	249	85.6	249	85.6	0.000	0.157
Preoperative pain	191	65.6	183	62.9	0.057	0.167
Unknown preoperative pain	28	9.6	32	11.0	0.045	0.043
No preoperative pain	72	24.7	76	26.1	0.032	0.206
Recurrent operation*	59	20.3	59	20.3	0.000	0.057

**Fig. 3:**
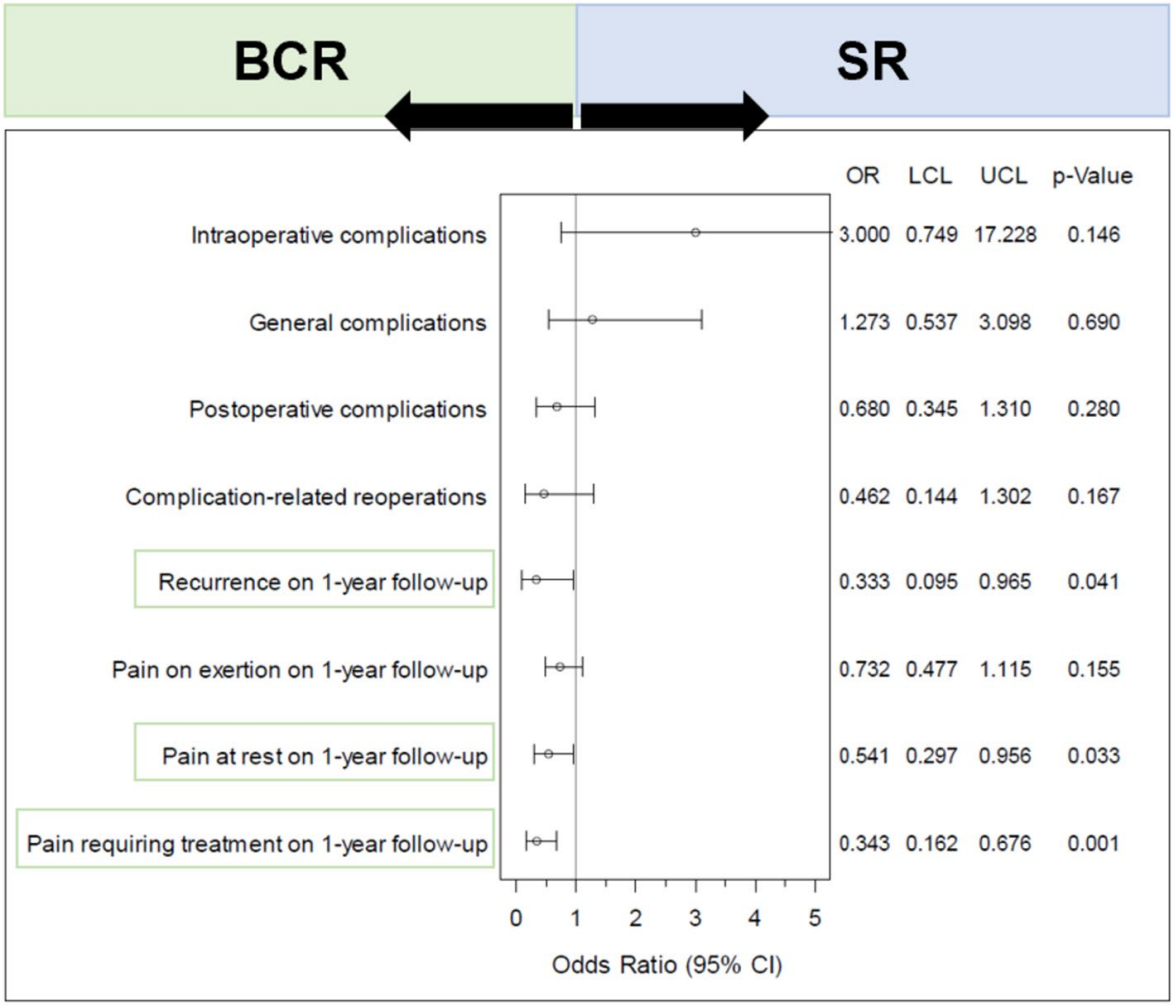
Forest plot—adjusted odds ratios (incl. confidence interval) for all outcome parameters when comparing: surgeries standard repair (SR) and considering biomechanically calculated repair (BCR) form the Herniamed registry

BCR show a significantly lower rate of recurrences, pain at rest, and pain requiring treatment at 1-year follow-up (Table 4; Fig. 3). Recurrence rates show a significant difference in favor of BCR surgeries (1.7% vs. 5.2%; *p* = 0.041, with no additional concordant cases; Table 5). This means that there are 1.7% matched pairs in which there is a recurrence after BCR surgery but no recurrence for the matched SR surgery. This contrasts with 5.2% recurrences without BCR consideration that do not appear with the matched BCR hernia. Thus, this is a systematic deviation in favor of BCR treatment. For pain at rest (6.9% vs. 12.7%; *p* = 0.033, with 1.37% concordant cases) and pain requiring treatment (4.1% vs. 12.0%; *p* = 0.001, with 0.34% concordant cases), there is a significant deviation in favor of the BCR. For all other outcome measures, no systematic deviation between the comparison groups could be shown (Fig. 3).

**Table 5:** Matched-pair results: proportion of pairs with complication/pain in both paired patients (concordant cases) and of those pairs with complication/pain in only one of the paired patients (disadvantageous cases), *n* = 291 matched pairs

	Concordant cases [%]	Disadvantageous cases [%]			OR for matched samples		
		BCR [yes]	BCR [no]	*p*-Value	OR	Lower limit	Upper limit
Intraoperative complications	0.00	*3.09*	*1.03*	0.146	3.000	0.749	17.228
General complications	0.69	*4.81*	*3.78*	0.690	1.273	0.537	3.098
Postoperative complications	0.69	*5.84*	*8.59*	0.280	0.680	0.345	1.310
Complication-related reoperations	0.00	*2.06*	*4.47*	0.167	0.462	0.144	1.302
Recurrence on 1-year follow-up	0.00	*1.72*	*5.15*	0.041	0.333	0.095	0.965
Pain on exertion on 1-year follow-up	3.78	*14.09*	*19.24*	0.155	0.732	0.477	1.115
Pain at rest on 1-year follow-up	1.37	*6.87*	*12.71*	0.033	0.541	0.297	0.956
Pain requiring treatment on 1-year follow-up	0.34	*4.12*	*12.03*	0.001	0.343	0.162	0.676

The forest plot in Fig. 3 illustrates the adjusted odds ratios for all outcome measures. The left side covers the surgeries with BCR and the right side covers the surgeries with SR. All outcome measures crossing the vertical line with their confidence interval indicate no conclusive effect for BCR or SR. If the confidence interval is entirely below 1, this indicates a significant disadvantage for operations without BSR consideration; if the interval is entirely above 1, this illustrates the disadvantage of considering BSR. The green boxes indicate significant differences.

## Discussion

Today, a wide range of reconstruction techniques for abdominal wall defects is available [28]. This facilitates a tailored approach but makes hernia care inconsistent with high recurrence rates [6]. Experienced surgeons tend to have better outcomes [9]. New combinations of reconstruction techniques are constantly being developed and promise further technical advances [6–8]. Repair based on biomechanical calculations is a new approach [12].

Large national registries such as Herniamed are essential to monitor and improve hernia care consistently. The Scandinavian countries have successfully demonstrated the use of national registries [29, 30]. The collection of large data sets on hernia surgery has shown that not one surgical approach is appropriate for every hernia repair [16]. While registries are useful for real-life data, they lack the consistency of randomized clinical trials. It should be noted that many questions about BCR cannot be fully answered at this time and will require future investigation.

The treatment of a hernia requires an individualized approach that is precisely tailored to the patient [16, 31]. For a reliable treatment, not only the size of the hernia, but also a potential unstable, debris-like zone around the hernia orifice must be taken into account. Anchoring a stabilizing mesh in an unstable area of the abdominal wall is likely to fail. Furthermore, the tissue extensibility and the level of stress are relevant. These factors can be assessed with a preoperative CT scan at rest and during the Valsalva maneuver (CTAV) [15]. CTAV provides additional information. First, it identifies unstable areas of the abdominal wall, that require additional support and are not suitable for mesh fixation. Second, it allows for an estimation of the individual patient’s tissue distension [15, 27]. This is important for a successful reconstruction as highly distensible tissue leads to a greater increase in the size of the hernia under stress and provides less stability to the abdomen [15]. A CT-Scan is particularly advisable mainly in complex cases, when there is a loss of domain or if a great tissue extensibility is suspected. It provides greater insight into the abdominal wall and contributes to improved outcomes. However, regardless of the CT scan, the BCR offers an improvement in outcome after 1 year of follow-up.

Hernia formation occurs early in the postoperative period, as indicated by a fascial dehiscence of more than 12 mm at 1 month after surgery [32]. We do not routinely look for such a significant fascial dehiscence 1 month after surgery [33]. However, complex reconstructions can have recurrence rates as high as 10% after 6 months [34]. After 43 months, 94% of all fascial distances greater than 12 mm had developed a manifest hernia [22].

An effective combination of techniques is required to meet the individual needs of the patient. As explained above, the GRIP/CRIP concept is based on the MDAR [1]. For several years, the ratio of the defect area to the mesh area is seen as crucial for a successful repair [13]. However, the meshes differ in their adhesion to the tissue and therefore require different fixations [26]. Also, the suture closure of the peritoneum and abdominal wall play an important role for the long-term durability of the reconstruction [24]. The GRIP/CRIP concept takes these factors into account to guide the surgeon through the jungle of reconstruction options while ensuring a safe outcome [15].

In many cases, the BCR leads to increased overlap and the selection of stronger material. This makes treatment safer, as this study and many others show [14, 35]. Interested hernia surgeons, including Herniamed participants, know the theory, and yet recurrence rates remain high in clinical reality. The BCR guides surgeons to use appropriate mesh size, safe materials, fixation, and techniques by providing them with a standardized algorithm. This improves hernia care. This propensity score matching is just a first step in demonstrating the major impact of biomechanically calculated reconstructions.

The use of propensity score matching in data analysis has limitations. Herniamed and its sub-registry, Stronghold, collect data in a prospective manner but without strict monitoring of data quality. Changes in patient data over time, such as weight, are not documented. Follow-up procedures rely on the efficiency of clinical practice. Timely and complete data collection was a mandatory for this PSM analysis. Analysis of the Herniamed and Stronghold data will allow us to examine effects that were anticipated and recorded. In the future, the unstable abdominal wall area, the magnitude and the distribution of tissue stretching, and the resulting stress–strain-relationships of a mesh repair may be of interest.

We believe that the BCR approach is particularly advantageous for the repair of large, complex, and recurrent hernias, as hernia size is a significant risk factor [36]. The surgeon was responsible for the selection of patients in our study. In the original sample, 20.6% or 23.9% of small hernias (< 4 cm) underwent SR or BCR, respectively. This could indicate the surgeons' learning curve in acquiring experience with incisional hernia repair using the BCR technique.

The following limitations have not been recognized yet. Significant numerical differences may not necessarily be clinically relevant. As the follow-up period increases, the clinical relevance of the results will become clearer. The frequency of recurrences will also become more apparent [37]. Over 3 years, the BCR has demonstrated superb durability with recurrence rates remaining below 3% [38]. We are close to obtaining the 5-year follow-up data, but we have not matched the propensity score yet. After 5 years, we expect the rate of recurrences to be 5% after BCR and 15% after SR [37].

## Conclusion

Biomechanical assessment and CT scans before surgery can improve patient care. Using the GRIP concept, over 99% of patients can be pain-free after 1 year with no recurrence [15, 27]. Surgeries with consideration of BCR had a significant advantage over those without BCR consideration in terms of recurrence, as well as pain at rest and treated pain. No significant difference was found between the comparison groups in terms of rates of complications, reoperations related to complications, or pain experienced during exercise in the follow-up period. Improving patient care can be achieved by taking into account the biomechanical factors at play.

## Data Availability

The raw data supporting the conclusions of this article will be made available by the authors, without reservation.
